# Effect of the PIVO Device on the Procedure of Phlebotomy from Peripheral IV Catheters

**DOI:** 10.1155/2018/7380527

**Published:** 2018-05-22

**Authors:** Suzanne Adams, Bridget Toroni, Meenal Lele

**Affiliations:** ^1^Thomas Jefferson University, Clinical Research Institute, 1015 Chestnut Street, Suite 317, Philadelphia, PA 19107, USA; ^2^Velano Vascular, Inc., 1028 Kearny Street, San Francisco, CA 94133, USA

## Abstract

Short peripheral catheters (SPC) are an existing conduit into many patients' veins and line draws from SPC are a desired method of routine blood collection especially in difficult venous access patients. The PIVO device facilitates blood collection through SPC and is being used clinically in a number of hospitals. This study aimed to determine the appropriate wait time following a flush and the minimum waste volume required to obtain an undiluted blood sample when using the PIVO device and how that differed from current guidelines from SPC line draws. A clinical study was conducted examining the analyte results of samples drawn with PIVO through a SPC at varying wait times following a saline flush. Both an initial waste volume and a postwaste sample were compared to a venipuncture control. The resulting samples showed no saline dilution as measured by sodium and creatinine results at all studied wait times. These findings suggest that blood collections using the PIVO device can produce a clinically valid sample with a 30-second wait following a SPC flush and no waste volume prior to sample collection.

## 1. Introduction

Line draws from short peripheral IV catheters (SPC) are a clinically desired method for blood collection because they can save patients pain and bruising, preserve veins, and eliminate the need to find a venipuncture site. However, because SPCs are used for infusions and are flushed with saline prior to blood collection, the utility of SPC draws for providing clinically valid blood samples has been questioned. Concern that an SPC infusion could dilute blood collected from the line, and the need to discard blood prior to sampling has historically prevented peripheral line draws from routine use in clinical practice.

However, a series of papers demonstrated the feasibility of obtaining valid samples from SPC line draws [1–7]. These studies compared blood collected directly from an SPC to a control venipuncture sample. The studies varied in the amount of blood wasted from the line and the amount of wait time between the flush and the draw. However, line collections were clinically equivalent to venipunctures in all studies. As such, line collections have become an acceptable procedure in some situations, though hemolysis rates from SPC samples remain higher than desired [8].

Allowing a sufficient wait time following an infusion or saline flush improved sample validity in 5 studies. Watson et al. (1983) and Zlotowski et al. (2001) agreed that a sample drawn from an SPC 2 minutes after an infusion showed minimal deviation from a comparison venipuncture [9, 10]. The Infusion Nurses Society (INS) 2016 standard used these results to recommend “infusing solutions should be stopped for at least 2 minutes prior to obtaining the blood sample [from a short peripheral catheter]” [11].

Wasting or discarding blood drawn from an SPC was also determined to affect validity of the subsequent blood sample. Baker et al. investigated the minimum volume of waste necessary to obtain an undiluted blood sample from an SPC following an infusion [12].

Analyte values from a venipuncture control were compared to those from an SPC draw following different waste amounts. Baker et al. established that, at minimum, an amount greater than the internal volume of the SPC and its attached extension tubing must be discarded (typically less than 1 mL) to prevent sample dilution [12]. Based on this result, the INS recommended 1-2 mL of waste before obtaining a sample from an SPC [11].

Recently, institutions have adopted the PIVO™ (Velano Vascular, Inc., San Francisco, CA) blood collection technology to collect blood samples via an SPC. PIVO is a single use device which threads a small, empty, sterile flow tube through the SPC into the vein to enable a blood collection. The PIVO device does not contain saline prior to blood collection; however PIVO is threaded through a saline locked SPC. In a previous study performed at the University of Pennsylvania [13] the PIVO device produced analyte results equivalent to those from venipuncture samples. In this study, the SPC was flushed with 5 mL normal saline 10 minutes prior to use. No tourniquet was used for the collections and one 4 mL tube was wasted prior to the analyzed sample.

Actual clinical use of PIVO differs from the procedure followed in the University of Pennsylvania study. The SPC is often flushed less than 2 minutes prior to PIVO use and a tourniquet may be used above the SPC to facilitate the collection. Given this variation in practice, the current study had two primary aims: (1) to determine the minimum required wait time following a saline flush of the SPC before using PIVO and (2) to determine if the necessary wait time following a saline flush could be reduced by wasting or discarding a tube of blood. In order to answer these questions, the study was designed to closely mimic the Baker et al.'s study, and the discard tube and the collection tube were compared to each other as well as to a control venipuncture.

## 2. Methods

The study enrolled 30 healthy adult volunteers recruited through the Jefferson Clinical Research Institute. Subjects were consented for participation in the study, and a brief medical history was performed to assess adherence to the inclusion criteria. Subjects were excluded if they had significant deformity of the arms, severe needle-phobia, morbid obesity, or hemolytic disorders. The study was reviewed and approved by the Thomas Jefferson University Office of Human Research Institutional Review Board.

Following consent, each subject had one 20-gauge SPC placed intravenously between the hand and the antecubital (AC). Following SPC placement and using a tourniquet, 4 PIVO samples were at varying wait times following saline flush (Table 1). Each PIVO collection obtained 2 tubes of blood. At the end of each PIVO draw, the device was removed and the SPC flushed with 5 mL normal saline. A 10-minute wait was used between each PIVO draw type. The order of the PIVO draws was alternated with each subject (Table 2). Table 1 lists the wait times for each draw type. The wait time listed was the time between the flush and application of the tourniquet on the upper arm above the SPC. The time to application of the tourniquet was used to limit variability and with the assumption that use of a tourniquet would stop or limit normal blood flow in the cannulated vein.

All subjects also had a control sample drawn by venipuncture in the arm opposite the SPC. To prevent bias caused by collection order, half of the study subjects underwent venipuncture prior to PIVO draws, while the other half underwent venipuncture following PIVO draws. Table 2 illustrates the sequence of study procedures for a sample of subjects.

In total, subjects had 9 tubes of blood drawn into 4 mL SST chemistry tubes (BD 367977 Vacutainer SST Tube with Hemogard Closure, 13 mm × 100 mm, 4.0 mL). Each tube was labeled to indicate the sequence (first tube versus second tube) and the draw type (control, A, B, C, and D). All tubes were immediately sent for analysis, and a full chemistry panel including a hemolysis index was reported. The laboratory used a Roche Cobas 6000 analyzer with 2 Component C501 chemistry analyzers. No subject follow-up was required; however, subjects were notified of any irregularities in the analyte results.

A two-part analysis was performed. For both analyses, specific focus was placed on the sodium and creatinine values. Dilution from a normal saline flush would increase sodium levels and decrease creatinine values. While all normal chemistry analytes were measured, focus was placed on these two analytes because of the direct effect saline dilution would have. A difference of least-square means analysis was used to compare the first tubes from each draw group to the control sample. This analysis was used to determine whether the first sample tube (waste tube) in each draw group was affected by the saline flush. If the sample was diluted, the analyte values would be different from the control and the null hypothesis of no difference would be rejected. The least-square means analysis was selected in order to more directly compare the results with Baker et al.'s work [12] which used a generalized mixed-model approach.

A second analysis was used to ensure that the second tube was equivalent to the control venipuncture sample. This analysis compared the second tube in each draw group to the control using Bland-Altman limits of agreement analysis. Limits of agreement analysis use the mean of the differences between two sets of samples and their standard deviations to calculate an upper and lower bound within which the true difference of means lies with a set 99% certainty. If the upper and lower bounds fall within the interval of difference created by predefined total allowable error, then the two methods of measurement can be considered equivalent. Acceptable performance was based on proficiency testing criteria for acceptable performance as established by CLIA methods used in clinical chemistry [14].

## 3. Results

Twenty-nine subjects completed the study and 27 subjects completed all blood collections. In 2 subjects, the fourth draw type using PIVO was not collected.  Table 3 provides information on patient demographics.

The differences of least-squares means analysis showed no difference between the first tube of any draw group and the control in either the sodium or creatinine values. By the lack of statistically significant differences in Tables 4 and 5 we can conclude that the two sample populations cannot be differentiated or are equivalent. When no saline flush was performed prior to PIVO use (Tube A1), there was no difference between the sodium and creatinine values in the venipuncture control versus the waste tube using PIVO (*p* > |*t*| 0.6417). The remaining comparisons showed that, following a saline flush, wait times as short as 30 seconds (group B1) did not impact the sodium and creatinine values of the first tubes. The acceptable variability in analyte results is designed to be smaller than the differences which would cause a change in treatment. As such, the lack of statistical difference between all samples implies that there would also be no significant clinical difference.

The Bland-Altman limits of agreement analysis similarly showed the second tubes of all draw groups to be statistically and clinically equivalent to the venipuncture control (Table 5). An additional analysis of other reported analytes and sample hemolysis showed that potassium values were also equivalent between all draw groups and venipuncture controls and that all samples were not hemolyzed.

## 4. Discussion

This study is built from the established research to determine best practices to avoid sample dilution when obtaining a blood sample using the PIVO device. This study specifically tested previous research that waste volume in SPC blood collections should be equal to or greater than the saline in the flow path and that a 2-minute wait is necessary to prevent dilution. The current analysis showed that no waste volume was required when using a PIVO device to draw a blood sample through a peripheral IV line. This analysis also established a shorter wait time requirement of 30 seconds, in contrast to prior wait time recommendations of 2 minutes [9, 10]. Past research established that a waste volume of 0.5 mL was needed to clear saline from an SPC line prior to blood collection [12]. The design of the PIVO device may explain this discrepancy. Unlike SPC catheters, which are typically saline locked with approximately 0.5 ml of saline in the catheter and tubing, the PIVO device enters the bloodstream with no saline in the blood collection flow path. In this sense, the PIVO device more closely resembles a venipuncture needle. As waste volume is not typically required when using a venipuncture needle for routine labs, no waste volume is required when using a PIVO device.

Differences in the required wait time between SPC and the PIVO device (2 minutes versus 30 seconds) may be partially explained by the characteristics of the SPC when the 2-minute wait time was established. One study examining wait time was performed in 1983 [9] using SPC technology that was very different from the catheter polymer materials and designs in use today. The most recent study to establish a 2-minute wait time was performed in 2001 [10]. This study, however, did not evaluate wait times less than 2 minutes, preventing a direct comparison with the current study.

This study was limited in the same ways as previous research in that it relied on healthy volunteers with recently placed SPC. Furthermore, like previous research, many of the study SPC were placed in the AC and as such the results may not be completely generalizable to SPC placed in particularly small veins such as those in the hand. That is, SPC placed in small veins with limited blood flow may have different characteristics than the ones studied here. However, blood flow rates in the index finger have been shown to be 1.5–7.1 cm/sec, implying that even in small veins of the hand, consistent blood flow will clear a vein of infusates in 30 seconds [15].

The 2016 INS guidelines [11] now recommend that SPC be considered for blood collection in certain populations. These include pediatric patients, adults with difficult venous access, presence of bleeding disorders, and the need for serial tests [11]. The ability to limit or reduce wait times between a SPC flush and blood collection using PIVO could significantly improve the workflow of completing SPC-based collection. The ability to reduce or eliminate waste volumes may aid in blood conservation strategies and reduce rates of iatrogenic anemia, especially as compared to collections from central venous access devices requiring 3–9 mL of discard [11].

## 5. Conclusions

These findings suggest that blood collections using the PIVO device can produce a clinically valid sample with a 30-second wait following a SPC flush and no waste volume prior to sample collection. This is the first study to examine the effect of using the PIVO device with methods that challenge the current procedure for collecting samples from an SPC. The results of this trial provide evidence supporting a procedure that reduces unnecessary blood waste and increases the feasibility of SPC-based blood collection.

## Figures and Tables

**Table 1:** Draw type and wait times.

Draw Type	Flush-Wait Step prior to start of PIVO procedure
A	0 mL Flush, 0-second wait
B	5 mL Flush, 30-second wait
C	5 mL Flush, 1 min wait
D	5 mL Flush, 2 min wait

**Table 2:** Sample sequence of study procedures.

Subject	Venipuncture	PIVO Draw	5 mL flush, 10 min wait	PIVO Draw	5 mL flush, 10 min wait	PIVO Draw	5 mL flush, 10 min wait	PIVO Draw	Venipuncture
1	X	A	X	B	X	C	X	D	
2		A	X	B	X	C	X	D	X
3	X	B	X	C	X	D	X	A	
4		B	X	C	X	D	X	A	X
5	X	C	X	D	X	A	X	B	
6		C	X	D	X	A	X	B	X

**Table 3:** Demographics.

Subject Characteristic	Value
Mean age (range)	34 (22–59)
Gender (%)	
Female	75
Mean BMI (range)	25.2 (20.5–32.8)
PIV Placement (%)	
Antecubital	83
Forearm	17

**Table 4:** Differences of least squares means, sodium/creatinine.

Tube	Estimate	Standard Error	Difference from Control	Standard Error	DF	*t* Value	*p* > |*t*|
Sodium							
A1	139.53	0.3494	−0.1247	0.2449	28	−0.51	0.6147
B1	139.55	0.3895	−0.1024	0.3169	28	−0.32	0.7490
C1	139.45	0.4510	−0.2059	0.3092	28	−0.67	0.5110
D1	139.51	0.3631	−0.1395	0.2762	28	−0.51	0.6175
Control	139.65	0.4461	- - - -	- - -	- - -	- - -	- - -

Creatinine							
A1	0.8133	0.02864	0.003280	0.01204	28	0.27	0.7873
B1	0.7897	0.03451	−0.02039	0.01623	28	−1.26	0.2194
C1	0.7828	0.03180	−0.02774	0.01507	28	−1.84	0.0763
D1	0.7988	0.02915	−0.01127	0.01476	28	−0.76	0.4514
Control	0.8100	0.02755	- - - -	- - -	- - -	- - -	- - -

**Table 5:** Bland-Altman limits of agreement analysis.

Sodium Comparison											
Test	Ref.	Test		Control		Difference (Test - Control)			B-A Limits of Agreement		Equivalent 4 mmol/L Allowable Error
		Mean	SD	Mean	SD	Mean	SD	*p*-value	LB	UB	
A2	Control	139.75	2.03	139.75	2.37	0.00	**1.54**	1.000	−3.02	3.02	Equivalent
B2	Control	139.46	2.15	139.75	2.37	−0.29	**1.61**	0.355	−3.44	2.86	Equivalent
C2	Control	139.61	1.93	139.75	2.37	−0.14	**1.51**	0.620	−3.10	2.81	Equivalent
D2	Control	139.74	2.35	139.82	2.39	−0.07	**1.75**	0.828	−3.51	3.36	Equivalent

Creatinine Comparison											
Test	Ref.	Test		Control		Difference (Test - Control)			B-A Limits of Agreement		Equivalent 5% Allowable Error
		Mean	SD	Mean	SD	Mean	SD	*p*-value	LB	UB	

A2	Control	0.82	0.15	0.81	0.15	0.01	0.07	0.602	−0.13	0.15	Equivalent
B2	Control	0.81	0.17	0.81	0.15	0.00	0.08	1.000	−0.15	0.15	Equivalent
C2	Control	0.81	0.15	0.81	0.15	0.00	0.06	1.000	−0.12	0.12	Equivalent
D2	Control	0.80	0.16	0.82	0.15	−0.02	0.08	0.161	−0.18	0.13	Equivalent
